# Shall I Show My Emotions? The Effects of Facial Expressions in the Ultimatum Game

**DOI:** 10.3390/bs12010008

**Published:** 2021-12-30

**Authors:** Sara Ferracci, Felice Giuliani, Alfredo Brancucci, Davide Pietroni

**Affiliations:** 1 Department of Neurosciences, Imaging and Clinical Sciences, Università degli Studi “G. d’Annunzio” Chieti—Pescara, 66100 Chieti, Italy; felice.giuliani@unich.it (F.G.); davide.pietroni@unich.it (D.P.); 2 Department of Motor, Human and Health Sciences, Università degli Studi di Roma “Foro Italico”, 00135 Rome, Italy; alfredo.brancucci@uniroma4.it

**Keywords:** ultimatum game, facial expressions, emotions, decision-making

## Abstract

Over the past fifteen years, research has demonstrated the central role of interpersonal emotions in communicating intentions, goals and desires. These emotions can be conveyed through facial expressions during specific social interactions, such as in the context of coordination between economic agents, where information inferred from them can influence certain decision-making processes. We investigated whether four facial expressions (happiness, neutral, angry and disgusted) can affect decision-making in the Ultimatum Game (UG). In this economic game, one player (proposer) plays the first move and proposes how to allocate a given amount of money in an anonymous one-shot interaction. If the other player (responder) accepts the proposal, each player receives the allocated amount of money; if he/she rejects the offer, both players receive nothing. During the task, participants acted as the responder (Experiment 1) or the proposer (Experiment 2) while seeing the opponent’s facial expression. For the responders, the results show that the decision was mainly driven by the fairness of the offer, with a small main effect of emotion. No interaction effect was found between emotion and offer. For the proposers, the results show that participants modulated their offers on the basis of the responders’ expressed emotions. The most generous/fair offers were proposed to happy responders. Less generous/fair offers were proposed to neutral responders. Finally, the least generous/fair offers were proposed to angry and disgusted responders.

## 1. Introduction

Some specific research over the past years has demonstrated the central role of emotions in communicating intentions, goals and desires [1,2,3]. These emotions, and the associated information, can be conveyed through facial expressions [3,4,5] during specific social interactions, such as in the context of coordination between economic actors, where information inferred from emotional expressions can influence certain decision-making processes during transitions, negotiations and agreements [2,6]. Negotiation is pervasive both in everyday interactions and (above all) in the business and management field [2]. In fact, the term negotiation refers to that shared decision-making process between parties with perceived divergent interests [7].

The first Ultimatum Game (UG) was developed forty years ago as a stylized representation of the final step of a negotiation, from Güth, Schmittberger and Schwarze [8]. In the UG, one player (proposer) plays he first move and proposes how to allocate a given amount of money in an anonymous one-shot interaction. If the other player (responder) accepts the proposal, each player receives the allocated amount of money; if he/she rejects the offer, both players receive nothing. According to the classical economic models, responders would have to accept any offer greater than 0 in order to maximize their payoff, since any offer is better than nothing, even if the offer is considered as unfair [8,9]. However, it was observed that participants tend to systematically reject unfair offers, namely those below the 30% of the total given amount of money [10,11], preferring to earn nothing rather than accepting an unequal distribution of resources.

Hence, there is a discrepancy between the classical theories, which assume rational decision making aimed at maximizing personal gain and utility [12], and the actual human behaviors, sensible to fairness and trust considerations, which form some of the basis of negotiation [11]. The UG is in fact an excellent way to measure people’s conflicts between gaining motivation and a sense of fairness, the same elements that are crucial in private and public negotiation.

### 1.1. The Intrapersonal and Interpersonal Effects and the EASI Model

In conceptualizing the informational value that an emotion can have during a negotiation, it is necessary to make a distinction between *intrapersonal* effects and *interpersonal* effects [13,14,15]. Most studies considered the effects of *intrapersonal* emotion, i.e., the influence that the negotiator’s affective state may have on his/her own judgments and behaviors [16]. In UG experiments, oftentimes only the emotions of the responder were considered, investigating the impact of his/her own affective state on the decision to accept or reject the offer [17]. 

Instead, the *interpersonal* approach investigates how the emotions of the expresser affect other individuals in a specific context. This approach was adopted by Van Kleef [18] who theorized how these emotions can provide strategic information about the expresser’s intentions, limits, priorities and preferences. These pieces of information affect social interactions and are relevant for the negotiation dynamics [15].

The two main ways through which interpersonal emotions can influence the decision-making during the negotiation process were always theorized by Van Kleef [19], who proposed the EASI model (Emotions As Social Information). The first way is the *strategic path*, through which the displayed emotions can be used to infer information related to the limits of the counterpart in order to modulate the proposals accordingly. On the other hand, there is the *affective path*, through which the expresser’s emotions can influence those of the opponent, arousing affective reactions based on social contagion. 

In this context, it is necessary to make a clear distinction between the results of the proposers’ behavior and the responders’ behavior. 

### 1.2. Effects of the Proposer’s Emotions on the Responder

In the literature, there are some studies where the participants take the role of the responder in order to investigate how the emotions of the proposer can influence the acceptance rate of an offer. Mussel and colleagues [5] manipulated the facial expressions of the proposer in a repeated UG, investigating the impact of either anger, happiness or neutral emotional expressions. It was found that the offers proposed with a smiling face were more often accepted compared to those made with a neutral facial expression, and that there were lower acceptance rates if the offers were made by a proposer with an angry facial expression. Liu and colleagues [20] highlighted how some emotions expressed by the proposer (such as sadness, disgust and fear) could increase the rejection rates of the responder. The authors explained their results by emphasizing that such negative emotions could have effects on fairness sensitivity by enhancing attention to the negative aspects of a specific situation. Mussel and colleagues [3] found that an offer made by a proposer with a happy expression was more likely to be accepted. The authors speculated that the positive feelings evoked by a happy emotional expression may in some cases mitigate perceived unfairness.

The responder’s behavior may in fact be more driven by perceived fairness, as well as by the affective path [19]. So, in this case, we can say that the characteristics of the proposer modulate the way responders respond to unfairness.

For instance, Pietroni and colleagues [21], adopting a modified version of the UG that contemplated a BATNA (i.e., Best Alternative To a Negotiation Agreement, an economic payoff that both parties could rely on in case of no deal), found that responders were willing to accept even severely unfair proposals (15 for them and 85 for the proposer) when fictitious proposers expressed happiness. However, this effect was mediated by the effect of inference regarding the BATNA from responders, namely that responders believed that proposers’ happiness signaled a better negotiation position, i.e., a rich BATNA.

This inference then led the responders to benevolently accept even predatory proposals insofar as they were perceived as fair with respect to the rich alternative payoff available to the proposer. This study was among the first to show that the proposer’s emotions can also influence the responder through not only an affective path but also through a strategic path [19]. In this way, the perspective on the effects of the proposer’s emotions was broadened, thus strengthening the interest in the study of the effects of the proposer’s emotions on the responder. 

This was one of the reasons motivating the present study which for the first time, as far as we know, carried out, at the same time, the investigation of the effects of the proposers’ emotions on the responder and vice versa, using exactly the same experimental paradigm.

### 1.3. Effects of the Responder’s Emotions on the Proposer

With regards to the studies that made the participant take on the role of the proposer, one of the pioneering contributions was by Van Dijk and colleagues [4], who investigated the effect of the responder’s happiness and anger on the offers subsequently formulated by the proposer. It was found that, in this case, the anger of the responder led the proposer to make a better offer, supposing that the counterpart could have a very high acceptance limit. In addition, in Lelieveld et al. [22], they examined how the expression of disappointment shown by the responder also modulated the offers expressed by the proposer. In Nelissen et al. [23], it was argued that offers in bargaining are driven by the emotions that proposers anticipate when contemplating their offers, making the final decision on the basis of anticipated guilt or fear.

These studies point out that the proposers are very careful to capture the emotional state of the responder, since they can use the information obtained to modulate their subsequent proposals.

From this point of view, since responders have the power of the last word, with their final decision to accept or reject the offer, they tend to be less motivated to follow a strategic path to infer information from the counterpart, namely the proposer [24]. On the other hand, proposers must consider not only their own outcome, but also the outcome of the counterpart and, therefore, they tend to be more interested in inferring strategic information to modulate their proposal [25]. For this reason, nearly all studies that considered the effects of interpersonal emotions focused on the role of the responder and neglected the interpersonal effects of the proposer’s emotions on the responder.

### 1.4. Emotions in Ultimatum Bargaining

In this context, some studies focused on the effects of some specific emotions, such as anger and happiness. Several theories suggest that anger plays a very important role in our interactions with others and that it can change our contractual position [17,26].

Research suggests, in fact, that anger is one of the most expressed negative emotions in bargaining [27]. According to a socio-functional analysis of emotions [13,19,28], this emotional expression conveys crucial information about the feelings, priorities and intentions of negotiators. It was observed, for example, that anger can promote different types of responses. In Lelieveld and colleagues [29], it was shown that, in the context of a negotiation, when anger was directed towards the offer, the participants used the information conveyed by this emotion to assess the limits of those in front of them, conceding more. On the contrary, if anger was directed to the person, it did not lead to higher concessions. In fact, when the context was an UG, anger expression could increase the propensity to reject offers, as a punishment behavior [30,31]. In particular, a lower acceptance rate of offers from proposers with an angry facial expression was also found [5]. On the other hand, happy facial expressions could promote trust and cooperation [30,32,33], leading to a higher level of acceptance of the offer when displayed during UG [5]. 

Another emotion that seems to be relevant in the UG is the emotion of disgust. It was observed that receiving unfair offers during an UG could lead to expressions of disgust, even without a core disgust elicitor [34,35], which in this case is indeed identified as moral disgust [36]. Disgust is closely related to the activation of the insula, a region of the brain that is also activated in response to unfair offers [36,37], suggesting that the violation of fairness and disgust could be associated [38].

### 1.5. Present Study and Hypotheses

This research aimed to investigate how the manipulation of the counterpart’s facial expression can affect the decision-making process facing the classic economic UG. To achieve this, we manipulated expressions on four levels: happiness, neutral, angry and disgusted. We then wanted to use the UG paradigm, an elegant model for investigating the interactions between counterparties [4], to understand how the crucial final phase of a modelled negotiation process could be influenced by the manipulated variables. To set up our paradigm, we took, as a reference study, the work of Mussel and colleagues [5]. Specifically, images of emotional faces were selected from a validated database [39] and were shown before each offer, playing, in turn, the role of the receiver and the role of the proposer. As a between design, each participant was randomly assigned to only one of the two conditions, namely proposer or responder. 

In our hypothesis, we expected that a smiling responder or proposer would be perceived as more friendly, thus leading the participant, in one case (as proposer) to make more generous offers, and in the other (as responder) to show higher acceptance rates. 

On the contrary, a responder or proposer with an angry or disgusted expression could be perceived in a more negative way, and could, therefore, lead the participant, in one case (as proposer) to make less generous offers, in the other (as a responder) to show lower acceptance rates.

We selected these specific emotions because among the interpersonal emotions during bargaining, anger and happiness have received the most attention [18,40,41].

We added the disgusted expression because it was observed that the emotion of disgust was strictly correlated with unfair offers in the UG [34,35]. Our aim was to investigate not only if the disgust induced in participants can influence their behavior, but also if observing expressions of disgust in opponents’ faces before proposing or accepting an offer could affect their behavior. Recent theories have shown that the emotional states that guide decisions tend to be stronger when they are congruent with those already present in the background [38,42,43]; therefore, our hypothesis was that the decisions to refuse unfair offers should be strongly associated with disgust and, therefore, because of the congruence effect, the facial expression of disgust should be more effective than anger in rejecting unfair offers.

The other novelty element of our study is that, to the best of our knowledge, this is the first study that compares the interpersonal effect of emotions considering, in the same study, both the effects of responders’ emotions on proposers’ allocation and the effects of proposers’ emotions on the responders’ decision to accept the offer using the same paradigm and the same facial expressions.

Finally, we explored the role of the individual attitude toward money as a possible moderator that could influence the generosity of the offer on the proposer’s role, and the acceptance rate in terms of the responder’s role. To this end, we used the Love of Money scale (LoM; [44]), a nine-item subscale of the Money Ethic Scale [45,46], which measures the level of importance that individuals attribute to money. Higher scores indicate that the individual craves money and attributes great importance to obtaining and holding it. The LoM was tested cross-culturally with respect to its measurement invariance in 29 countries [47]. A previous study [48] found that LoM positively correlates with the emotional attachment that individuals attribute to physical banknotes, demonstrating a tight link between attitude toward money and perception of actual monetary value. Since higher LoM scores indicate a greater attachment to money, we hypothesize that individuals may be less generous in their offers when they are in the role of proposer. However, when those individuals are in the role of responder, they may be more willing to accept an offer, even when it is unfair, since every offer implies a monetary gain.

## 2. Experiment 1—Responder

In the first experiment, all the participants were assigned to the role of the responder.

### 2.1. Materials and Methods

#### 2.1.1. Participants

A total of 113 participants were involved in the experiment. Among them, we excluded 12 participants who did not correctly complete the comprehension test concerning the UG (see details in the Procedures section). Therefore, 101 participants were included in the analyses (61 women; *M* age = 25.8, SD = 13.4). 

The adequacy of the sample size was determined by an a priori power analysis using G*Power (version 3.1.9.4). In our analysis, we took into account the effect size reported by Mussel et al. (2013) concerning the main effect of emotional expression on the acceptance rate of the proposer’s offer (η^2^ = 0.1). Therefore, we selected F-tests and ANOVA (repeated measures and between factors) in G*Power and entered the following parameters: effect size f(U) = 0.33; α = 0.05; Power (1 − β) = 0.95; number of groups = 1; number of measurements = 4. The minimum sample size required was *N* = 54. 

All the participants gave written and informed consent before beginning the experiment and were naïve about the real purposes of the study. 

#### 2.1.2. Stimuli

We selected the images of emotional faces from a validated database [39]. Specifically, the emotional facial expressions were manipulated on 4 levels: happiness, neutral, angry and disgusted. We selected a total of 48 images of 48 individuals (24 female faces and 24 male faces). For each of the 48 individuals, four pictures were used: a happy one, a neutral one, a disgusted one and one with an angry facial expression. The 48 stimuli were subdivided into 4 lists, so that the 4 facial expressions of each individual were randomly assigned to one of the lists. Using this method, in the role of proposer, each individual made only one offer to each participant with only one of the 4 possible facial expressions.

#### 2.1.3. Procedures

The test was administered using Qualtrics^©^ survey software. Before starting the actual test and after they entered their age and gender, the participants were instructed on the UG rules, then they were told that the computer randomly assigned him/her the role of responder. Before starting the actual experiment, participants responded to a comprehension test concerning the rules of the UG. They were asked the result of a hypothetical question regarding the splitting of a sum of money, choosing among three possible outcomes. Only those who correctly answered this question were included in the analyses. 

**Figure 1:**
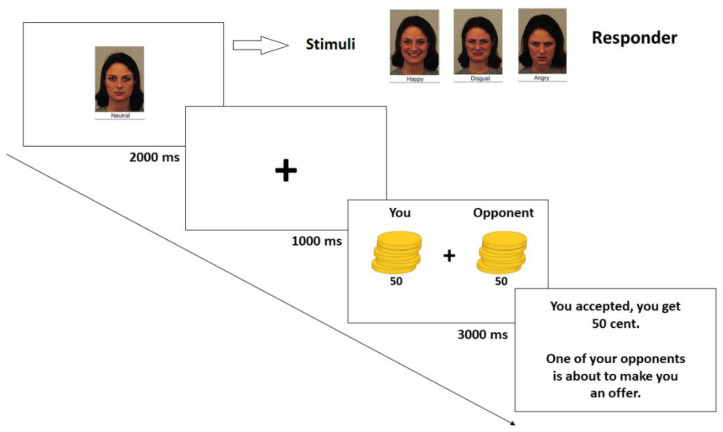
Task timeline for the ultimatum game in the responder’s experiment.

Each participant played as a responder in a series of 48 one-shot trials. In particular, each trial began with the picture of the proposer (see Figure 1), and after a fixation cross (500 ms), the offer was displayed. The sum of money to split was always 1 € [49]. We manipulated the fairness level of the offers as follows. The group of fair offers was: 60 cents (Overly Fair; [5]), 50 cents (Fair) and 40 cents (Moderately Fair). The group of unfair offers was: 30 cents (Moderately Unfair), 20 cents (Unfair) and 10 cents (Extremely Unfair). Each fictitious proposer made only one offer to the participant with only one of the 4 possible expressions. In addition, each individual was always linked to only one of the possible offers (10–90, 20–80, 30–70, 40–60, 50–50, 60–40) regardless of the assumed expression. The order of the 48 offers was randomized for every participant, who could decide whether to accept the displayed offer or not. After every interaction, feedback was displayed in order to inform the participants about how much they received and how much the proposer received based on the selected option. After that, the sentence “One of your opponents is about to make you an offer” was shown on the screen, before the beginning of the following trial. At the end of all interactions, each participant completed the Love of Money (LoM) scale, a 9-item subscale of the Money Ethic Scale [45,46] developed by Tang [44,47]. For each item, participants answered using a 5-point Likert scale (ranging from 1 to 5) and the score was obtained by averaging all the responses (min = 1, max = 5). The M score of our participants was 3.52 (SD = 0.71)

### 2.2. Data Analysis 

For each participant, the rejection of the offer was coded as 0, whereas its acceptance was coded as 1 [5]. Therefore, the Dependent Variable (DV) was an index, ranging from 0 to 1, that measured the average proportion of accepted offers, or the average acceptance rate, where 0 indicates that the offer was always rejected, whereas 1 indicates that the offer was always accepted. 

Data analysis was performed using Statistica 8.0 software (StatSoft) and the significance threshold was set at *p* = 0.05. The first analysis was a repeated measures analysis of variance (ANOVA), with Emotion (anger, disgust, neutral, happiness) and Offer (10, 20, 30, 40, 50, 60) as within factors. 

The second analysis was a mixed ANOVA with Emotion (anger, disgust, neutral, happiness) and Offer (10, 20, 30, 40, 50, 60) as within factors, Gender as a between factor, and Age and LOM as covariates [48]. 

### 2.3. Results

**Figure 2:**
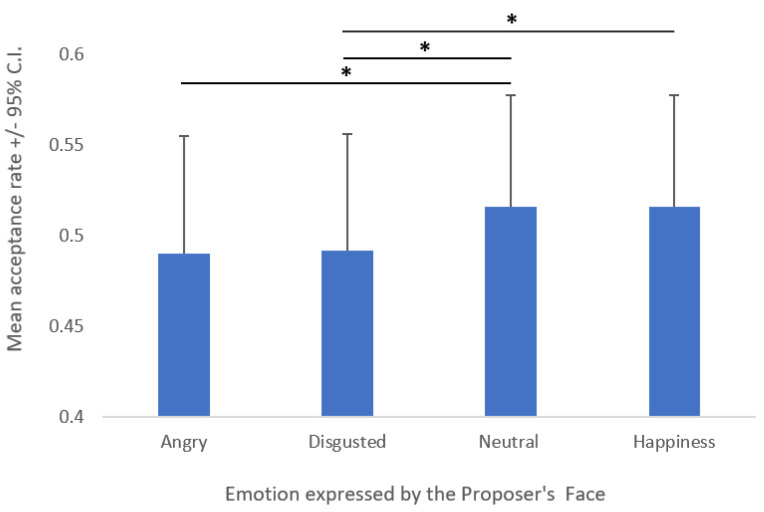
Main effect of the emotion expressed by the proposer’s face on the mean acceptance rate. Asterisks (*) indicate significant post hoc comparisons.

The first ANOVA showed a main effect of Emotion (F (3, 246) = 2.99; *p* = 0.032; *η*^2^*_p_* = 0.036; Figure 2). Tukey’s post hoc comparison revealed that angry expressions led to a lower average acceptance rate compared to neutral expressions (*M* = 0.49 vs. 0.52; *p* = 0.042), whereas disgusted expressions differed from both neutral (*M* = 0.49 vs. 0.52; *p* = 0.035) and happy expressions (*M* = 0.49 vs. 0.52; *p* = 0.042); all *p*-values are reported in Table 1. 

**Figure 3:**
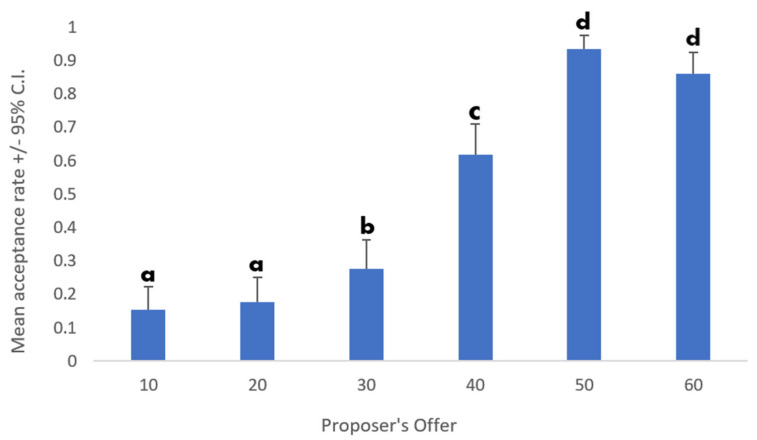
Main effect of the proposer’s offer on the mean acceptance rate. Different letters indicate significant differences.

Moreover, the main effect of Offer was also significant (F (5, 485) = 13.85; *p* < 0.001; *η*^2^*_p_* = 0.12; Figure 3). The interaction between Emotion and Offer was not significant (F (15, 1455) = 0.61; *p* = 0.87; *η*^2^*_p_* = 0.006). All *p*-values are reported in Table 2. The effect of Gender was not significant either (F (1, 97) = 1.07; *p* = 0.3; *η*^2^*_p_* = 0.04). The second ANOVA showed the same main effects of Emotion and Offer. Moreover, the following effects were detected: Age as a covariate was significant (F (1, 97) = 3.97; *p* = 0.049; *η*^2^*_p_* = 0.039), as well as the interaction between Age and Offer (F (5, 485) = 3.18; *p* = 0.008; *η*^2^*_p_* = 0.032), and the interaction between LOM and Emotion (F (3, 291) = 4.12; *p* = 0.007; *η*^2^*_p_* = 0.041).

In order to investigate the effect of the covariates, post-hoc regression analyses were performed. The first series of regressions had the average acceptance rate of the six offers as DVs, and Age as the predictor. The results indicated that the willingness to accept the extremely unfair offer increased with age (*β* = 0.23; *p* = 0.017; *R*^2^ = 0.042). No other significant effects were found.

The second series of regressions had the four Emotions as DVs, and LOM as the predictor, and no significant effect was detected.

## 3. Experiment 2—Proposer

In the second experiment, all the participants were assigned to the role of the proposer.

### 3.1. Materials and Methods

#### 3.1.1. Participants

A total of 134 participants were involved in the experiment. Among them, we excluded 19 participants who did not answer correctly to the comprehension test concerning the UG (see details of Experiment 1 in the Procedures section). Therefore, 115 participants were included in the analyses (65 women; *M* age = 28, SD = 6.58). 

All of them gave written and informed consent before beginning the experiments and were naïve about the real purposes of the study. 

#### 3.1.2. Stimuli

The selected images were the same as those already used in Experiment 1. In Experiment 2, the selected faces represented the responders. The facial expression was manipulated on the same 4 levels: happiness, neutral, angry and disgusted. The same 48 images of 48 individuals were used (24 female faces and 24 male faces). For each of the 48 individuals, four pictures were used: a happy one , a neutral one, a disgusted one and lastly one with an angry facial expression. The 48 stimuli were always subdivided into 4 lists, so as to randomly assign the 4 facial expressions of each individual to one of the lists. Each list contained an equal number of males and females.

#### 3.1.3. Procedures

The test was administered using Qualtrics© survey software. Before starting the actual test and after they entered their age and gender, participants were instructed on the UG rules, then they were told that the computer randomly assigned him/her the role of proposer. Before starting the actual experiment, participants responded to a comprehension question concerning the rules of the UG (see Experiment 1).

**Figure 4:**
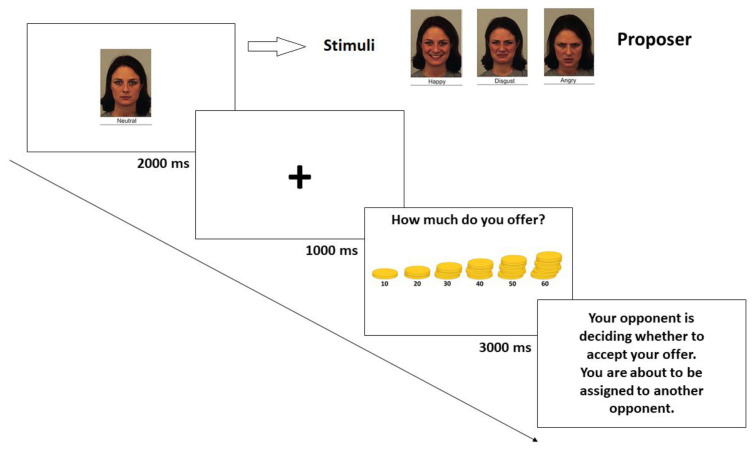
Task timeline for the ultimatum game in the proposer’s experiment.

Each participant played as a proposer in a series of 48 one-shot trials. In particular, each trial began with the picture of the responder (see Figure 4) and, after a fixation cross (500 ms), the offers were displayed. The sum of money to split was always 1 € [49]. In the role of proposer, the participant had to select an offer from the available choices (10, 20, 30, 40, 50, 60; [5]). Each responder appeared with only one of the 4 possible expressions. After every interaction, no feedback was provided, in order to avoid the implementation of some type of strategy [49], and instead, the sentences “Your opponent is deciding whether to accept your offer. You are about to be assigned to another opponent” were displayed before the start of the following trial. At the end of all the interactions, each participant completed the Love of Money (LoM) scale, a 9-item subscale of the Money Ethic Scale [45,46]. For each item, participants answered using a 5-point Likert scale (ranging from 1 to 5) and the score was obtained by averaging all the responses (min = 1, max = 5). The M score of our participants was 3.6 (SD = 0.69).

### 3.2. Data Analysis 

For each participant, the average offer corresponding to each responder’s expression was calculated. Data analysis was performed using Statistica 8.0 software (StatSoft) and the significance threshold was set at *p* = 0.05. The first analysis was a one-way analysis of variance (ANOVA), with Emotion (anger, disgust, neutral, happiness) as a single within factor.

The second analysis was a mixed ANOVA with Emotion (anger, disgust, neutral, happiness) as a within factor, Gender as a between factor, and Age and LoM as covariates.

### 3.3. Results

**Figure 5:**
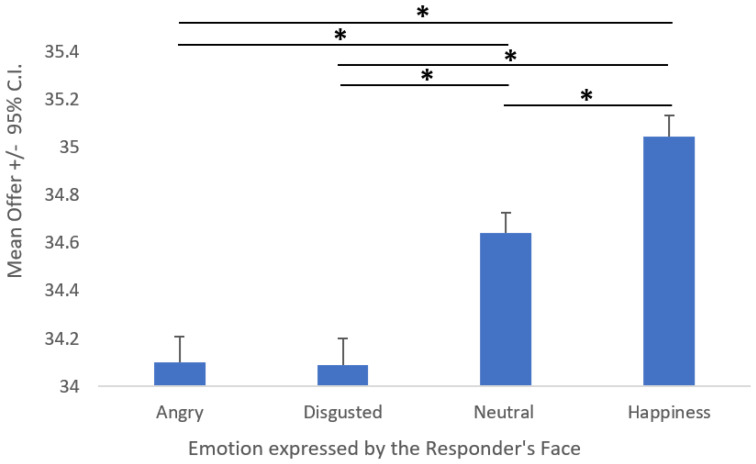
Main effect of the emotion expressed by the responder’s face on the proposer’s average offer. Asterisks (*) indicate significant post hoc comparisons.

The first ANOVA showed a significant effect of Emotion (F (3, 288) = 52.025; *p* < 0.001; *η*^2^*_p_* = 0.35; Figure 5). Tukey’s post hoc comparison revealed that angry and disgusted expressions led to a similar average offer (*M*~34.1; *p* = 0.9), whereas neutral expression led to a higher average offer (*M* = 34.6; *p* < 0.001), and happiness led to the highest average offer (*M* = 35; *p* < 0.001).

The second ANOVA showed no main effect of Emotion nor of Gender. However, the effect of both covariates was significant: Age (F (1, 111) = 5.3; *p* < 0.023; *η*^2^*_p_* = 0.05); LoM (F (1, 111) = 5.66; *p* < 0.019; *η*^2^*_p_* = 0.05). Finally, there was a significant interaction between Age and Emotion.

**Figure 6:**
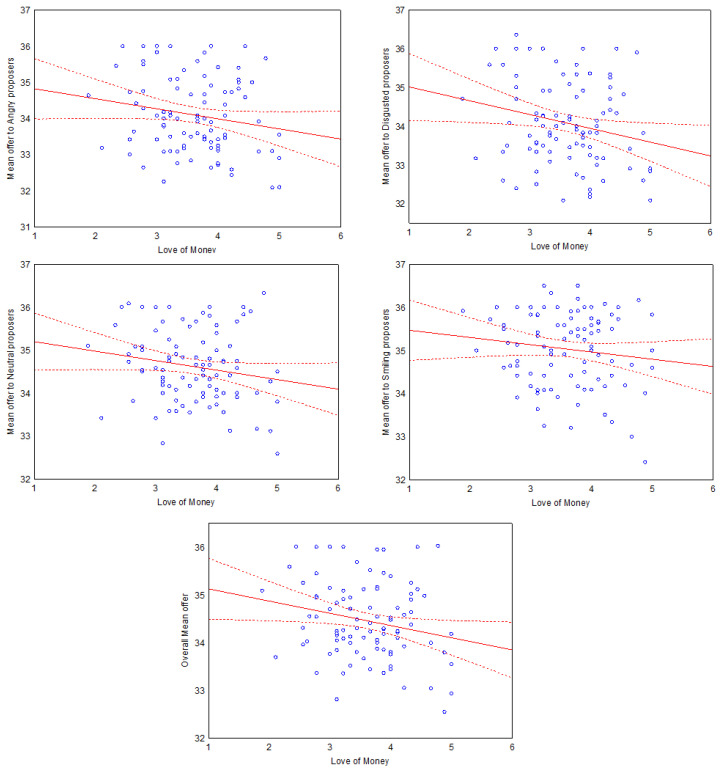
Correlation between Love of Money score and mean offer in different proposers’ emotional conditions. From **top left**: anger, disgust, neutral, happiness and overall mean offer. Dashed lines denote 95% confidence interval.

In order to investigate the effect of the covariates, post-hoc regression analyses were performed. The first series of regressions had LoM as a continuous predictor and the following average offers as DVs: angry, disgusted, neutral, happiness and overall average offer. The results revealed that the overall average offer decreased as the LoM score increased. The same pattern was found for angry, disgusted and neutral expressions, but not for smiling expressions. See Table 3 for the complete statistics and Figure 6 for the scatterplots.

The second series of regressions had Age as a predictor and the following average offers as DVs: angry, disgusted, neutral, happiness and overall average offer. The results revealed that the overall average offer decreased as the age increased. The same pattern was found for angry and disgusted expressions, but not for neutral and smiling expressions. See Table 4 for the complete statistics.

## 4. Discussion

Emotional expressions represent a decisive aspect when it comes to social interactions, interpersonal communication and coordination. During these interactions, the emotional manifestations conveyed by specific facial expressions provide clues on the internal state of the counterparts, on their most probable behavior [50] and on their limits and expectations (e.g., [4,51]). Furthermore, in the context of bargaining, we tend to pay close attention to the emotions expressed by our opponents because they could also provide valuable information on their subsequent moves [4]. A recent study also demonstrated how the proposer’s emotion can be strategically used to infer the opponent’s bargaining position, consequently affecting the responder’s acceptance rate [21].

Compared to previous studies (e.g., [3,5]), we wanted to investigate, through the same experimental paradigm, both the role of proposer and the role of responder. 

### 4.1. Responder

For the responder (Experiment 1), our results show that the decision was strongly driven by the fairness of the offer, as was expected in this game. There was also an effect of emotions: neutral emotion and happiness led to higher acceptance rates compared to anger and disgust. However, this effect was much smaller than the fairness effect. Moreover, no interaction effect was found between the emotion expressed by the proposer and the offer’s fairness. On the contrary, in the reference study, Mussel and colleagues [5] found a larger main effect of emotions and a rather small interaction between fairness and emotion. In particular, the offers from proposers with smiling facial expressions were more often accepted than those from proposers with neutral facial expressions. They also found lower acceptance rates for offers from proposers with angry facial expressions and, more generally, elevated rejection rates that were a function of the fairness of the offer.

Some theories have attempted to explain irrational behaviors in fairness-related decision making. One of these is the “inequity aversion” theory, which confirms individuals’ preferences for fair outcomes [52,53]. Another theory that can be referred to is the “reciprocity equilibrium” theory, which holds that rejection in the UG could be interpreted as a kind of social punishment that aims to promote fair offers in subsequent bargaining, to establish a good reputation and to enforce norms of fairness [54,55]. 

For this reason, the interpersonal effect of the proposer’s emotion on the responder might be attenuated by perceived fairness. In fact, the possible information conveyed by proposers’ emotions cannot be used at a strategic level to modulate the responder’s decision since he/she only has the power to decide whether to accept or reject the offer [56], unless a BATNA is involved [21].

Therefore, according to the EASI model [19], and from the responder’s perspective, we should only expect that he/she will be influenced by the emotions of the proposer through a solely affective path, which can influence his/her mood and benevolence [21]. 

We can speculate that the proposer’s expression of emotions before the responder decides to accept or reject an offer can have an impact on responders’ sensitivity to unfairness. Specifically, smiling proposers may compensate for the unfairness of the offer. 

### 4.2. Proposer

The results of our second experiment show instead that proposers modulated their offers on the basis of the expressions they saw on the responders’ faces. Specifically, anger and disgust had no differential effects, but they were perceived as similarly negative, disconfirming our initial hypothesis regarding the higher impact of disgust. However, more generous offers were proposed to responders with neutral expressions compared to the two negative ones and even more generous offers were proposed when the responders showed happy facial expressions rather than either angry or disgusted expressions, but also compared to neutral facial expressions.

Even though these effects had already been observed in the literature, confirming that emotions in general play a crucial role in negotiation contexts [14,18,41,57], there are disagreements and inconsistencies in evidence related to interpersonal mechanisms, specifically underlying the expressions of anger and happiness, which have differential effects depending on the object or person toward which they are directed [2] and on the characteristics of the context in which they are expressed [58]. 

In the work of Van Kleef and colleagues [18,41], for example, communicating anger during a negotiation task led the counterpart to make larger concessions. On the contrary, concessions tended to be reduced when the opponent communicated happiness. The authors hypothesized that the emotion expressed, whether happiness or anger, was used by the counterpart to infer the opponent’s limits. If the opponent was angry, he/she was perceived as having ceiling limits and, therefore, the negotiators placed less ambitious demands and made larger concessions, trying to reduce the risk of rejection. If, on the other hand, the opponent was happy, he/she was seen as having lower limits and, therefore, the counterpart made smaller concessions [15,18,41].

The same effect was observed in the research of Sinaceur and Tiedens [40], in which participants tended to concede more to an angry opponent during face-to-face negotiation, attributing high-power to the counterpart that displayed negative emotion [59].

Therefore, in the cases reported above, it seems that communicating anger turns out to be beneficial in negotiation. However, in other cases, it has been noted that communicating anger can be counterproductive, as it could also decrease the degree of cooperation during a negotiation. In the work of Van Dijk et al. [4], for example, it was observed that communicating anger became detrimental when the counterparts reciprocated the emotions of their opponents. The authors explained this aspect in terms of emotional contagion [4,60], in which it was essentially observed that participants became angry when confronted with an angry opponent, leading to competitiveness, avoidance and rejection [5,61,62], and they became happy when confronted with a happy opponent, leading to trust and cooperation [3,5,63].

One of the proposals that was made in the literature, and that attempted to reconcile these divergent results, was first proposed by Steinel and colleagues [51]. They argued that the different effects observed with these emotions could depend on the target toward which they are directed. They can in fact be directed either to the offer proposed or to the bargainer as a person. It follows that when the emotion is directed to the offer, it can be inferred as a strategic information helping to predict the opponent’s limits [29] and, therefore, it can lead to greater concessions in the case of anger, and vice versa in the case of happy expressions. On the contrary, if the same emotions are instead directed to the person, and are not directly connected to the offer, expressing anger can lead to negative results, because it indicates poor cooperation, while expressing happiness can be favorable in this case, because it can inspire trust and encourage cooperation.

This interpretation appears to be in line with our results. In terms of how the presented paradigm was structured, the faces that expressed a certain emotion always appeared before any offer and, therefore, the emotions were found to be directed to the counterparts themselves. Each trial began, in fact, with a picture of the proposer or with a picture of the responder and then, after a fixation cross, the offer was displayed. This led, in the cases in which our participants assumed the role of proposer, to greater concessions being made to happy or neutral counterparts, rather than to angry or disgusted ones. 

Taken together, our results show not only that the responders are susceptible to fairness [8,9,64], but also that the proposers can express irrational behaviors linked to fairness [56], giving a preventive punishment to those whom he/she assumes could behave in an unfair and uncooperative way, which is inferred from an emotional expression that is associated with negative behaviors. Similarly to the responder, therefore, the proposer seems willing to sacrifice his/her payoff to punish those who appear uncooperative and unreliable.

Compared to the responder, who has the power of the last word [24], the proposer has to make a proposal considering the outcome of both him/herself and the responder, evaluating to what extent the rope can be pulled [11] and may, therefore, be more interested in understanding the other’s emotions.

According to the EASI model [19], we should in fact expect, in the case of the proposer, that he/she would be more motivated by the strategic path that allowed him to infer information about the responder’s emotions and to modulate his/her allocation accordingly. 

Furthermore, an exploratory analysis on the proposer, using Age as a covariate, showed that with increasing age, the mean offer decreased, but only because of negative expressions. We could explain this unexpected result by considering some literature that suggests that older participants more often choose the more rational economic strategy [65,66], which, from the proposer’s point of view, means offering less.

### 4.3. The Hypothesis of Disgust

Another hypothesis that we wanted to test was the effect of the expression of disgust. It was observed that this emotion is closely related to unfair offers within the UG [34,35]. In particular, in a 2003 study [36], Sanfey and colleagues used fMRI to examine some participants while playing the UG and found that an unfair offer received during an interaction activated distinct regions of the anterior insula, an area that, it was previously noted, can be activated in cases of both physical disgust (due, for example, to smell or taste; [67]) and emotional disgust, due to moral disgust [35]. Our aim was, therefore, to verify whether observing expressions of disgust before proposing or accepting the offers could change the behavior of the participants in relation to the offers themselves. Our hypothesis in this regard was that the expression of disgust is perceived in a more negative way than the expression of anger. However, as noted in the second experiment, anger and disgust are not different from each other, but are perceived as equally negative, refuting our initial hypothesis regarding disgust. One of the reasons that we did not find this difference could be that many affective and cognitive functions besides disgust are associated with activation of the insula, including anger [34]. In other contexts, in fact, disgust was proven to be significantly different from other negative emotions, such as sadness. In a particular study carried out by Moretti and Pellegrino [38], it was shown that, compared to being in a sad or neutral mood, feelings of disgust significantly increased the rejection rates of unfair offers. This shows that, although sharing the same negative valence, disgust seems to have a stronger impact on the subsequent decisions implied in the UG dynamics compared to sadness. The authors addressed this difference, claiming that while feeling disgust implies some sort of action, in order to avoid something revolting [38,68], sadness is a more passive emotion correlated to loss and that, for this reason, it appears to be less driving in decisions and actions related to UG. Anger, on the other hand, seems to share with disgust not only the insula activations and the negative valence, but also the willingness to act and to react to the unfair offer that may occur during UG [17], and this can be a factor underpinning why we did not observe substantial differences between the two emotions in the subsequent outcomes and decisions within the game.

### 4.4. The Effect of LoM

Our last hypothesis was that LoM would modulate both the proposer’s and responder’s decisions. Regarding the responder, our hypothesis was not confirmed.

However, regarding the proposer, our hypothesis was confirmed since the LoM score was negatively correlated with the average offer. This trend was observable in every emotion condition, but it was not significant for smiling expression. This result suggests that higher attachment to money leads to a strategy that maximizes the possible monetary outcome, offering less in order to keep more money for themselves. However, this strategy is also riskier since it exposes the proposer to a responder’s possible rejection. This pattern of choice is coherent with the idea that higher LoM individuals are more risk tolerant [69]. 

Finally, considering the single emotion conditions, even though the correlation was only significant in the disgust condition, the other conditions exhibited a close trend toward significance. Therefore, we did not consider this effect reliable enough to be discussed here, but rather a possible hint to develop further hypotheses in this regard. 

### 4.5. Limitations and Future Direction

One limitation that must be highlighted is the use of hypothetical stakes in both of our experiments. In fact, it is possible that the difference in effect size between our Experiment 1 and the results obtained by Mussel and coworkers [5] was due to the fact that the latter provided small economic incentives to participants. Specifically, we found a small effect size, whereas Mussel and coworkers [5] reported a medium effect size for the same main effect. Future research should investigate the same topic with real and higher incentives, in order to evaluate if this can modulate the outcome in some way. 

Another limitation that we should consider is that participants had been exposed to a greater proportion of negative stimuli than positive ones, which may have impacted the results.

## 5. Conclusions

In summary, our results corroborate the idea that while the responders rely more on the offer’s size to make their own decisions, the proposer, having to take into account the outcome of both parties, takes into high consideration the emotions expressed by the responders as a source of strategic information to understand how to modulate his/her proposal. In fact, when the one who is negotiating does not have clear information about the limits of his/her counterpart at his/her disposal, emotions can represent a strategic source from which to draw in order to understand the limits, the intentions and the interests of the person in front of him/her. It has been noted that positive emotions lead to more generous offers and improve cooperation, while, on the contrary, negative emotions lead the counterpart to propose more unfair offers even with the risk of sacrificing their own payoff. This tendency is even more accentuated in individuals who attribute great relevance to money. The UG constitutes a clear and simple coordination context through which it is possible to study different bargaining dynamics, the results of which, however, can also be applied to real negotiation contexts. In this case, our study suggests that positive feelings can lead to more cooperative behaviors and fair agreement.

## Figures and Tables

**Table 1:** *p*-values relative to Tukey’s post hoc comparisons between the four emotions and the acceptance rate in all the experimental conditions.

	Angry	Disgusted	Neutral	Happiness
**Angry**		0.999	0.042	0.051
**Disgusted**	0.999		0.034	0.042
**Neutral**	0.042	0.034		0.999
**Happiness**	0.051	0.042	0.999	

**Table 2:** *p*-values relative to Tukey’s post hoc comparisons between the offers and the acceptance rate in all the experimental conditions.

	10/90	20/80	30/70	40/60	50/50	60/40
**10/90**		0.999	0.018	<0.001	<0.001	<0.001
**20/80**	0.999		0.056	<0.001	<0.001	<0.001
**30/70**	0.018	0.056		<0.001	<0.001	<0.001
**40/60**	<0.001	<0.001	<0.001		<0.001	<0.001
**50/50**	<0.001	<0.001	<0.001	<0.001		0.391
**60/40**	<0.001	<0.001	<0.001	<0.001	0.391	

**Table 3:** Regression results: LoM as a continuous predictor and angry, disgusted, neutral, happiness and overall average offer as DVs. Asterisks (*) indicate significant post hoc comparisons.

Mean Offer (DVs)	*β*	*P*	*R* ^2^
Angry	−0.22	0.02 *	0.04
Disgusted	−0.24	0.03 *	0.05
Neutral	−0.19	0.04 *	0.03
Happiness	−0.13	0.16	0.009
Overall	−0.24	0.01 *	0.05

**Table 4:** Regression results: Age as a continuous predictor and angry, disgusted, neutral, happiness and overall average offer as DVs.

Mean Offer (DVs)	*β*	*P*	*R* ^2^
Angry	−0.25	0.01 *	0.06
Disgusted	−0.30	0.001 *	0.09
Neutral	−0.18	0.05	0.02
Happiness	−0.006	0.9	0.001
Overall	−0.24	0.01 *	0.05

## Data Availability

The data that support the findings of this study are available from the corresponding author, upon request.
